# A Proposal for a Process from as Low as Reasonably Achievable to an Ultra-Low-Level Goal in Chest Computed Tomography

**DOI:** 10.3390/jcm13164597

**Published:** 2024-08-06

**Authors:** Isabelle Fitton, Etienne Charpentier, Emina Arsovic, Jennifer Isaia, Manon Guillou, Aurélien Saltel-Fulero, Laure Fournier, Claire Van Ngoc Ty

**Affiliations:** 1 Department of Radiology, Georges Pompidou European Hospital, Paris Cité University, APHP, 75015 Paris, France; etienne.charpentier@aphp.fr (E.C.); emina.arsovic@aphp.fr (E.A.); jennifer.isaia@aphp.fr (J.I.); aurelien.saltel-fulero@aphp.fr (A.S.-F.); laure.fournier@aphp.fr (L.F.); claire.vanngocty@aphp.fr (C.V.N.T.); 2 PARCC UMRS 970, INSERM, 75015 Paris, France

**Keywords:** computed tomography, ultra-low dose, ALARA, process, optimization

## Abstract

**Background/Objectives:** To define and evaluate a radiation dose optimization process for chest computed tomography (CT) imaging. **Methods:** Data from unenhanced and enhanced chest CT acquisitions performed between June 2018 and January 2020 in adult patients were included in the study. Images were acquired on a Siemens SOMATOM^®^ Definition Edge CT. Dose values, including Dose.Length Product (DLP) and Volume CT Dose Index (CTDI_vol_), were collected. Low doses (LDs, 25th percentiles), achievable doses (ADs, 50th percentiles), and diagnostic reference levels (DRLs, 75th percentiles) were calculated before and after parameter modifications. A process was defined and applied to patient data. For unenhanced chest CT, data were differentiated according to three groups: high dose (HD), optimized dose (OD), and ultra-low dose (ULD). Dosimetric changes between protocols were expressed as mean CTDI_vol_ % (CI95%). A Mann and Whitney statistical test was used. The diagnostic quality score (DQS) of a subset of 70 randomly selected CT examinations was evaluated by one radiologist. The DQS was scored according to a three-point Likert scale: (1) poor (definite diagnosis impossible), (2) fair (evaluation of major findings possible), and (3) excellent (exact diagnosis possible). **Results**: Data were collected from 1929 patients. For unenhanced chest CT protocols, only one process loop was run. A dose comparison between the chest CT protocol before the use of the process and the three groups showed a decrease of −38.3% (9.7%) and −93.4% (24.2%) for OD and ULD, respectively, and an increase of +29.4% (4.7%) for HD. For the enhanced chest CT protocol, two optimization loops were performed, and they resulted in a mean dose reduction of −50.0% (2.6%) compared to the pre-optimization protocol. For all protocols, the DQS was greater than or equal to 2. **Conclusions**: We proposed a radiation dose optimization process for chest CT that could significantly reduce the dose without compromising diagnosis.

## 1. Introduction

Over the past decade, the concept of dose optimization, known as the As Low As Reasonably Achievable (ALARA) principle, has emerged as a tool for evaluating professional practices [1,2] and protecting patients from unnecessary radiation exposures. The approach is mainly based on the comparison of local median doses to national and international diagnostic reference levels [3] (DRLs) for a few clinical indications [4,5] or acquisitions. It is a level of investigation used to detect abnormally high radiation doses and to determine if acceptable image quality could be achieved at lower doses. A procedure for the analysis of DRLs has been defined by the International Commission on Radiological Protection (ICRP) in the form of an audit cycle [3]. This cycle includes a comparison with national or international benchmarks, and it must be carried out every three years.

The DRLs have been defined for the most common CT examinations, such as chest CT, for patients of similar weights and heights [6]. The value of chest CT has been demonstrated in the literature for the surveillance and screening of pathologies and is therefore widely used [7,8,9]. International studies have been conducted to define the DRLs for chest CT for pulmonary embolism detection, lung cancer screening and follow-up, and chest CT with and without contrast injection [4,6,10] (Supplementary Materials, Table S1). 

The ALARA principle, when applied, should constrain the local median radiation doses to respect those DRLs. However, compliance with national and international guidelines is not sufficient and should not result in centers avoiding additional optimization. Indeed, DRLs are updated slowly relative to technological developments and consequently are quite often significantly higher than clinical practice. As a result, the comparison with DRL misses opportunities for optimization in most patients outside of the range of standard-weight adult patients [11]. Another limitation is the underlying, unrealistic assumption that the CT scan pool is technically homogeneous at national and international levels. Moreover, the types of examinations or procedures specified for the published DRLs may not be directly relevant to a particular practice. Finally, in most dose surveys, image quality is assumed to be acceptable without a documented assessment by a radiologist [12], which is nevertheless decisive for declaring a successful CT examination and should remain the first goal. To address these drawbacks, which can greatly limit the relevance of DRLs, Rehani [12] introduced the notion of an acceptable-quality dose, based on averaged dose values, which can be set for each facility, including images of clinically acceptable quality and defined according to weight groups. However, a continuous dose reduction strategy based on a radiation dose target as low as possible for an acceptable diagnostic quality is not included in that approach.

Based on these findings, the aim of this study was to define and evaluate a method for stepwise optimization of the radiation dose delivered during chest CT scans.

## 2. Materials and Methods

### 2.1. Population

This retrospective monocentric observational study was conducted from June 2018 to January 2020 in the emergency department of the Georges Pompidou European Hospital in Paris, France. Patients aged under 18 years were excluded. The protocol was approved by the Scientific and Ethical Committee of our institute (IRB authorization number: 00011928) and registered by the French National Data Protection Agency (Reference number: 20220210145855); the need for patient consent was waived. 

### 2.2. Factors Influencing Radiation Dose in CT Examinations

Images were acquired using a CT scanner single-source split-filter dual-energy CT system (Siemens Healthineers SOMATOM^®^ Definition Edge). The Volume Computed Tomography Dose Index (CTDI_vol_) was used as a metric to compare the radiation dose delivered by the scanner between protocols and to assess the gain in radiation dose following changes in acquisition or reconstruction parameters. The acquisition parameter settings were set in the user interface that specified the scan.

The detector configuration for all chest CT examinations in this study was 128 × 0.6 mm. A smaller total beam collimation would have a higher CTDI_vol_ for the given detector width per data channel. The pitch value is the table feed per gantry rotation divided by the beam width. It can be changed in increments of 0.05 to adjust the total scan time. The exposure time per rotation takes into account the gantry rotation time and angular acquisition range, and it can also be modified. In both cases, however, modulations automatically adjust system parameters to maintain dose and image quality. Tube current modulation consists of XYZ modulation. This means that the tube current is modulated in both the angular and longitudinal directions. The image quality reference parameters, Quality Reference mAs (QRM) and Reference kVp (Ref. kVp)), define the desired level of image quality, and any change will have an effect on the CTDI_vol_; a decrease in the QRM will result in noisier images but at a lower dose (Supplementary Materials, Figure S1). The tube potential is not modulated in the same way as the tube current; it does not change with different tube positions around the patient and only works in conjunction with tube current modulation. CTDI_vol_ will be approximately proportional to the square of the percentage change in Ref. kVp. QRM and Ref. kVp settings are defined for a standard-sized adult patient weighing 75 kg to achieve a specified image quality. The modulations can be adjusted according to clinical needs by varying a cursor position from 1 to 12. For non-contrast scans, it should be set to 3, and for CT angiography, a setting of 11 is recommended by the manufacturer.

### 2.3. Process Definition

**Figure 1:**
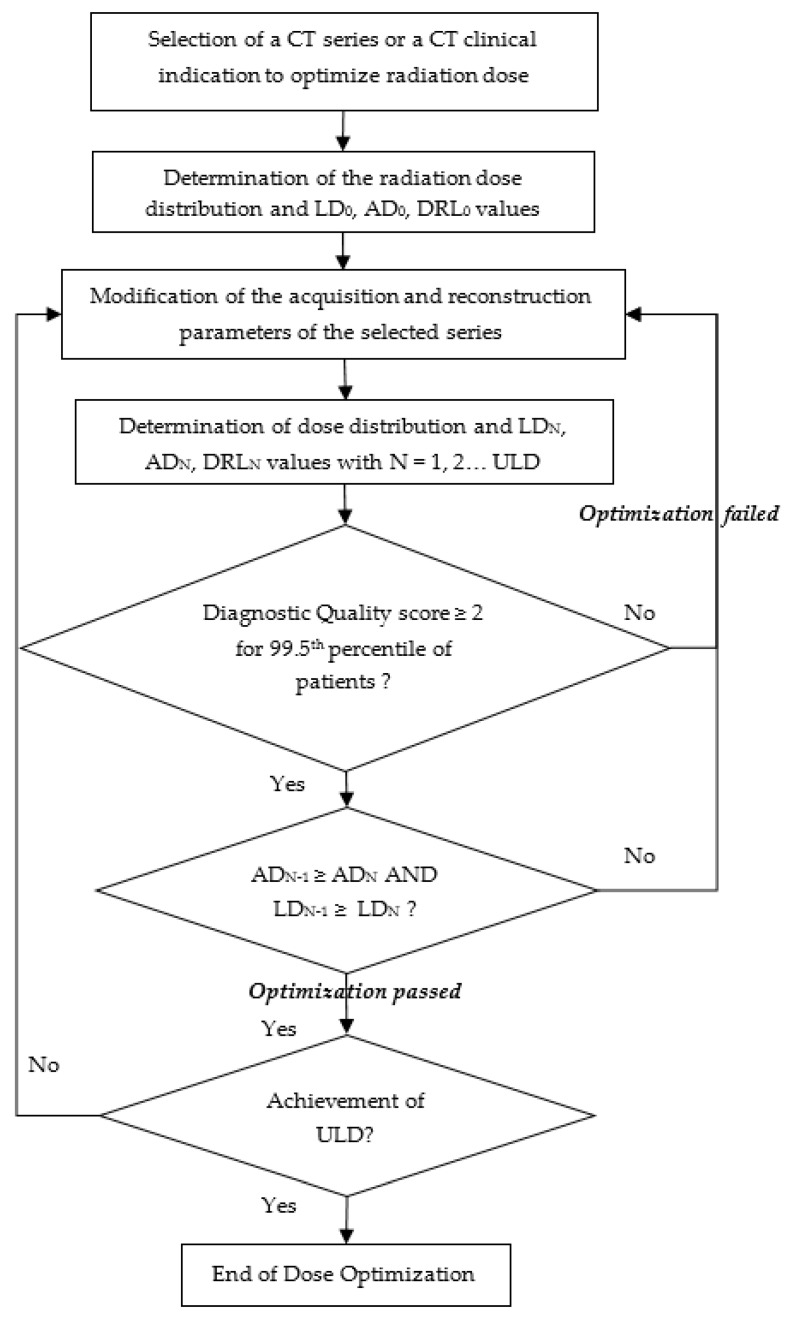
Radiation dose optimization process in chest computed tomography. Note: the LD, the AD, and the DRL are the 25th, 50th, and 75th percentiles of the distribution of median values of all dose data, respectively. N represents the step level in the process. Abbreviations: LD: low dose; AD: achievable dose; DRL: diagnostic reference level; ULD: ultra-low dose.

A radiation dose optimization process was defined with an ALARA goal (Figure 1). It was based on the CTDI_vol_ and Dose.Length Product (DLP) parameters, expressed in low dose (LD, 25th percentiles), achievable dose (AD, 50th percentiles), and local diagnostic reference level (DRL, 75th percentiles), combined with the diagnostic quality constraint. It consists of several stages. The starting point was the selection of the series or clinical indication for optimization and the clinical context or patient morphotype. The second step was to calculate the initial values for LD, AD, and DRL, noted as LD_0_, AD_0_, and DRL_0_, respectively; these values started the process. Next, the scan and reconstruction parameters were modified and applied to patients during several weeks. At this point in the process, the LD, AD, and DRL values, noted as LD_1_, AD_1_, and DRL_1_, respectively, were again determined and had to satisfy three conditions to validate the optimization loop: the diagnostic image quality score performed on a patient sample had to allow the radiologist to make a clinical characterization of the pathology in accordance with the clinical task for 99.5^th^-score image patients, AD_1_ should not exceed AD_0_, and LD_1_ should not exceed LD_0_. If these conditions were met, the process could continue with a change in scan parameters, the LD_2_, AD_2_, and DRL_2_ values were calculated, the process loop continued, and it was re-evaluated at each stage of the process.

### 2.4. Implementation of the Process in Chest CT Protocols

- Differentiation of unenhanced chest CT protocol by patient morphotype or clinical context

In the case of the unenhanced chest CT, the standard protocol prior to optimization was used as a reference. This was split into three different chest protocols: high-dose (HD), optimized-dose (OD), and ultra-low-dose (ULD) chest CT. Image acquisitions covered the first to the last thoracic vertebrae. No intravenous contrast was administered. 

The HD protocol was specifically designed for patients who were overweight or obese, with a body mass index (BMI) greater than 30 kg.m^−2^. For the HD chest CT protocol, the standard chest CT protocol has been modified by increasing the maximum value of the kilovoltage modulation system to 140 kVp instead of 120 kVp [13]. This represents an increase of just over 20%. 

The OD protocol applies to all standard-sized patients presenting to the emergency department for a chest CT scan. The OD chest CT protocol was defined as a 51% reduction in the CTDIvol from 6.78 mGy to 3.30 mGy by decreasing the QRM and Ref. kVp by 20%. 

Finally, the ULD protocol was defined more specifically for screening for lung cancer, for pulmonary patients undergoing repeated CT scans during their medical follow-up, or for patients with a BMI less than 28.5 kg.m^−2^. Images were acquired at fixed 100 kVp and 10 mAs to obtain a CTDI_vol_ close to 0.4 mGy according to phantom results [14]. The tube current and kilovoltage modulation systems were disabled for the ULD chest CT protocol only. 

For all unenhanced chest CT protocols, raw data were reconstructed using level 3 of the ADvanced Modeled Iterative REconstruction (ADMIRE) algorithm with the medium-smooth mediastinum reconstruction kernel I31f and level 4 with the medium-sharp lung reconstruction kernel I50f. All acquisition and reconstruction parameters for the three protocols are detailed in Table 1. Only one iterative optimization loop was run.

- Iterative process cycles for optimization of enhanced chest CT protocol

In the case of the enhanced chest CT, two iterative optimization loops were performed. The first loop, N_1_, was started with the standard pre-optimization protocol as the reference, N_0_. Optimization consisted of moving the tissue cursor position from 9 to 11 and increasing the slice thickness from 0.6 mm to 1 mm.

In the second loop, N_2_, while keeping the parameters of the first loop the same, the Ref. kVp was reduced from 100 to 80 kVp.

For all enhanced chest CT protocols, raw data were reconstructed using level 3 of the ADMIRE algorithm with the medium-smooth mediastinum reconstruction kernel I26f and level 4 with the medium-sharp lung reconstruction kernel I50f. 

All acquisition and reconstruction parameters for the three protocols are detailed in Table 2.

### 2.5. Image Quality Evaluation

The diagnostic quality was evaluated by one radiologist with 5 years of experience blinded to the radiation doses and the CT protocol used on a Syngovia^®^ workstation (Siemens Healthcare). In total, 70 chest CT examinations were analyzed in terms of diagnostic quality (DQS), including 40 chest CT scans without contrast injection, with 10 each of HD, OD, ULD, and reference protocols, and 30 chest CT scans with contrast injection, including 10 each for the N_0_, N_1_, and N_2_ loops of the process. The overall DQS was scored according to a 3-point Likert scale [15]:

(1) Poor: Definite diagnosis impossible. A value equal to 1 was considered unsatisfactory for clinical use.
(2) Fair: Assessment of the most significant findings and considered the minimum expected diagnosis for a given clinical task in the process. Minor artifacts or limitations that were unlikely to affect diagnostic confidence.
(3) Excellent: Exact diagnosis possible, with no artifacts or limitations. Scans were considered for diagnosis if they had a DQS greater than or equal to 2.

### 2.6. Statistical Analysis

Dose values including DLP and CTDI_vol._ were collected from the Dose Management System (DMS) (RDM, v.1.4.6.1, Medsquare, Paris, France). Quantitative variables were expressed as 25th, 50th, and 75th percentiles or the median [Q1; Q3]. The mean CTDI_vol_% (CI95%) was used to express dosimetric changes between protocols. A Mann–Whitney test was used to compare doses. 

## 3. Results

**Figure 2:**
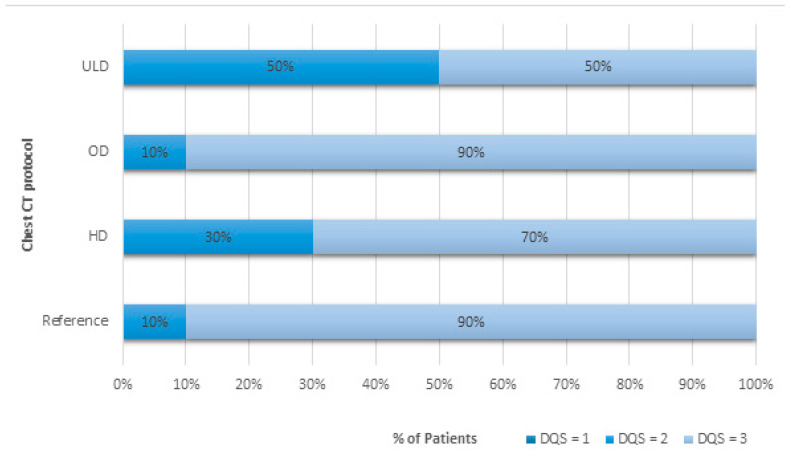
Diagnosis quality scoring (DQS) in percentages for unenhanced chest CT protocols differentiated by patient morphotype or clinical context. Abbreviations: ULD: ultra-low dose; OD: optimized dose; HD: high dose.

**Figure 3:**
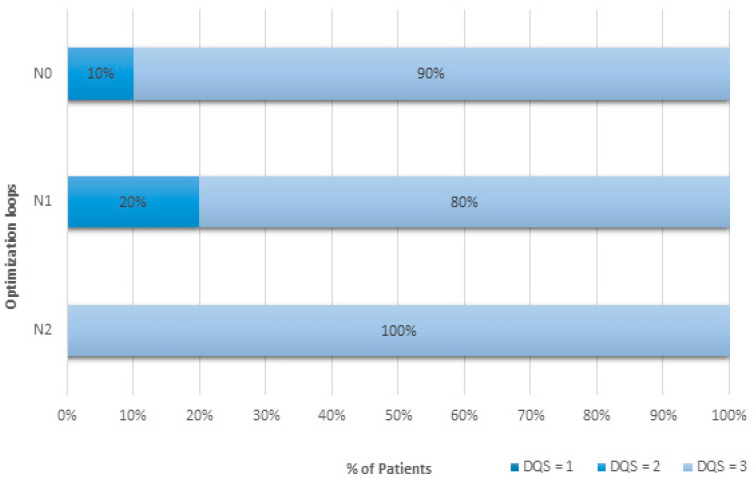
Diagnostic quality scores (DQSs) in percentages for multiple process optimization loops for a contrast-enhanced chest CT protocol. Abbreviations: N represents the step level in the process.

CT examinations from 1929 adult patients were studied. All patient sizes were included. CTDI_vol_ and DLP values are shown in Table 3 and Table 4. The diagnostic quality scores are provided in Figure 2 and Figure 3.

The DQS of 2 was due to a respiratory artifact for one patient in the reference protocol and noise in the LD protocol case. The three conditions of the optimization process were met in the case of patients with a standard morphotype (Figure 1). First, all had DQS scores greater than or equal to 2. Second, the AD of the optimized dose protocol was 3.7 mGy. This was less than the reference protocol AD of 6.3 mGy. And, third, the LD of the optimized dose protocol was 2.7 mGy, which was less than the reference protocol LD of 5.0 mGy. Adherence to process dose requirements regarding CTDI_vol_ was also successfully achieved for DLP values. So, the optimization process could continue.

### 3.1. Differentiation of Unenhanced Chest CT Protocol According to Patient Morphotype or Clinical Context

For unenhanced chest CT protocols, 865 patient data were reviewed for the radiation dose. 

For the OD protocol, a reduction in Ref. kVp from 120 to 100 reduced significantly mean doses by—38.3% (9.7%). DLP values were significantly lower with the OD chest CT protocol at 131 [99; 189] mGy.cm compared with the reference protocol with DLP at 222 [178; 285] mGy.cm (Table 3). CTDI_vol_ values were also significantly lower for the optimized-dose chest CT protocol compared to the reference protocol, decreasing from 6.3 [5.0; 8.0] mGy to 3.7 [2.7; 5.3] mGy, respectively. The DQS was scored greater than or equal to 2 for 10% of examinations and equal to 3 for more than 90% of examinations for both protocols (Figure 2). 

The definition of a specific HD chest CT protocol for obese patients allowed for achieving a DQS of 2 in 30% of examinations and a score of 3 in 70% of CT examinations, but at the significant expense of dose increases up to 299 [214; 361] mGy.cm compared to the reference chest CT protocol of 222 [178; 285] mGy.cm (Table 3 and Figure 2). 

CTDI_vol_ values were also significantly higher for the HD chest CT protocol compared to the reference protocol, increasing from 6.3 [5.0; 8.0] mGy to 8.3 [5.7; 10.6] mGy, respectively: +29.4% (4.7%). A score of 2 was explained by either discomfort due to respiratory artifacts or image noise in one of the patients. As a reference for the “standard” patient, the DRLs of unenhanced chest CT acquisition are 350 mGy.cm and 545 mGy.cm for DLP and 9.5 mGy and 15 mGy for CTDI_vol_ according to French [12] and US [6] data, respectively. Therefore, our results for the OD chest CT protocol and even for the HD chest CT protocol were still below international DRLs.

In the case of the ULD CT protocol, the overall quality of chest images was classified as good with a definite diagnosis possible in 50% compared to 90% by using the reference chest CT protocol (Figure 2). All ULD CT examinations had sufficient DQS to allow for evaluation of important findings while significantly reducing the radiation doses by 93.4% (24.2%) to 15 [14; 16] mGy.cm and 0.4 mGy for DLP and CTDI_vol_, respectively, compared to the reference protocol (Table 3). A score of 2 was explained by discomfort from image noise.

### 3.2. Iterative Process Cycles for Optimization of Enhanced Chest CT Protocol

Radiation dose data from 1064 patients were used to evaluate the iterative process. The results are shown in Table 4 and Figure 3.

The first optimizing loop (N_1_) failed to meet all optimizing criteria. Although DQSs were greater than or equal to 2 for every patient, dose criteria were missed for one of two conditions (Figure 3). Indeed, the LD value expressed in DLP at the N_1_ level of the process was 176.1 mGy.cm. It was superior to the LD value of the N_0_ level, which was equal to 174.1 mGy.cm. In contrast, the analysis based on CTDI_vol_ met the two dose criteria: the AD CTDI_vol_ in N_1_, equal to 5.7 mGy, was less than the AD in N_0_, equal to 8.1 mGy, and the LD CTDI_vol_ in N_1_, equal to 4.5 mGy, was less than the LD in N_0_, equal to 5.0 mGy (Table 4). The optimization process failed only when using the DLP, even if dose reduction between N_0_ and N_1_ levels was −25.6% (2.9%) and statistically different. Other parameters had to be changed.

The process continued by switching from loop N_1_ to loop N_2_, changing the acquisition parameters. At N_2_, the condition for image evaluation was met: all examinations were evaluated with a quality of 3 (Figure 3). These results show that the image quality was improved by +10% and +20% compared to the results obtained at the N_0_ and N_1_ levels of the process, respectively. Regarding the dose, process conditions were met (Table 4). The AD for CTDI_vol_ at the N_2_ level of the process was 3.8 mGy. This was less than the AD value for the N_1_ level, which was 5.7 mGy. The LD value at the N_2_ level was 3.1 mGy, which was lower than the LD value of 4.5 mGy at the N_1_ level. DLP values fulfilled these dosimetric requirements as well. Dose reduction was statistically different between N_0_ and N_2_ levels: −50.0% (2.6%). These results validated step N_2_ of the process and allowed for continuation of the process.

There was a statistically significant decrease from N_0_ to N_1_ and from N_1_ to N_2_ for both dose metrics. Comparison with international DRLs showed that our results, even before optimization, were well below what could be expected from a chest CT with injection: 596 mGy.cm and 16 mGy for DLP and CTDI_vol_, respectively [6].

## 4. Discussion

A method was developed and evaluated to optimize the dose delivered to chest CT adult patients in routine clinical practice. This process should help to determine the next goal for achieving a major dose reduction while maintaining diagnostic value. The process was based on three dose indicators, LD, AD, and DRL, which are the 25th, 50th, and 75th percentiles, respectively, of the distribution of the values of all dose data. The introduction of the 25th percentile parameter into the process tends towards an ultra-low dose as the ultimate process goal, adapted to each scanner technology. These indicators were constrained by conditions that validate or reject the various process optimization loops. It also relies on the radiologist’s diagnostic quality score. A minimal requirement on an expected diagnostic level defined as the evaluation of major findings was included, which allowed for constraining the process for a clinical task. For the enhanced chest CT protocol, using two loops of the process resulted in a dose reduction of −50%. The process satisfies the need to gradually optimize protocols over time and may integrate technological or software developments or test other configurations of acquisition/reconstruction parameters.

AD and DRL are commonly used for radiation dose evaluation in the literature [4,6,16]. The comparison of our pre-optimization AD values with the national and international DRLs has shown that these benchmarks are high. Satisfying these criteria is not an end in itself and should not be used as an argument to stop trying to optimize protocols. Several authors have already specified [11,12] the drawbacks of DRLs, and the process showed in this study makes them possible to overcome as a method applicable to every CT device regardless of their technological performance and for all patients whatever their weight. Each radiology department would thus be able to apply it to a specific patient profile or protocol and define its own objective in dose reduction step by step. 

This method can be applied to any scanner, regardless of its technological capabilities. Optimization process requires a precise understanding of the technological possibilities, as well as the influence of variations in the acquisition parameters of the scanner concerned on image quality and dose.

The optimized radiation dose parameters were established from clinical data. They were not determined from oversimplified phantom data. Indeed, the major limitation of optimization studies based on phantoms is the failure to take into account the heterogeneity and complexity encountered in the human body and organs [17]. However, phantom tests are an important preliminary step in assessing the impact of parameter variation on image quality and noise and in avoiding the need to carry out such tests on patients. Once identified, these parameters must be validated in a limited number of patients before being used in all patients. 

In this study, we were particularly interested in thoracic CT scans. Indeed, this examination plays a central role in the management of patients with severe respiratory symptoms and is frequently used [8,9,18]. The approach adopted in the dose optimization process distinguished between different cases based on patient morphology and repeatability for monitoring lung pathology over time. 

For the special cases of screening or long-term follow-up, or for patients with BMIs less than 18.5 kg/m^2^, the process differentiated an ultra-low-dose protocol. The dose of this ULD protocol was drastically reduced by −93.4% compared to the reference unenhanced chest CT protocol, while maintaining sufficient image quality for diagnostic purposes. The results could be further improved in terms of detectability through the use of tin filtration [14]. In lung cancer screening, it is particularly important to define and evaluate the performance of an ultra-low-dose protocol to keep the doses delivered during the examination as low as possible. The image quality of the ultra-low-dose protocol was rated by the radiologist as lower than that of the reference protocol due to the presence of noise in the image. However, new deep-learning image reconstruction technologies are now reducing the noise to deliver increasingly lower radiation doses and improved image quality [19,20]. Further research into the ULD chest CT protocol that produces high-quality, low-dose images could help make lung cancer screening available to more people [7] and reduce cumulative radiation doses for patients who must undergo follow-up scans over many years, such as lung transplant patients [9].

This process also established an obese HD chest CT protocol. Indeed, the increase in the incidence of obesity in children and adults over the past few decades has created several new challenges for CT examinations, including those of the chest [21]. Images of these patients can appear noisy. To achieve diagnostically acceptable image quality, applying the ALARA principle to this patient profile means delivering a higher dose than for standard patients. The mean dose increase was +29.4% compared to the reference chest CT protocol. However, all CT scans were interpretable. For this reason, the case of obese patients has been considered separately, as the issue requires special management in terms of acquisition CT protocols and adapted contrast injection protocols [22]. 

The inclusion of patients with different morphologies, especially obese patients, limited the dosimetric metrics we could use. In fact, the process was based on the analysis of DLP and CTDI_vol_. An alternative would have been to use a radiation dose metric that would have taken into account the patient’s body size and the X-ray-attenuation properties of the examined tissue, such as the size-specific dose estimate (SSDE) [23]. However, the determination of the SSDE requires the measurement of diameters, such as the patient’s anterior–posterior diameter and the lateral diameter from a CT localization or an axial image. In obese patients, the patient’s entire body may be out of the field of view, making these measurements impossible. Further research into the determination of the SSDE should allow for the removal of the need to measure the patient’s diameter, thus allowing the SSDE to be used in the process [24]. 

The process allows for step-by-step optimization to ensure diagnosis, and it can integrate the continuous development of new technologies and image reconstruction methods in CT, with the expectation of further reductions in radiation dose levels [25]. Indeed, newer post-processing technologies, such as iterative reconstructions algorithms, including projection-data-based [26], model-based [27], and combined hybrid and prior-projection-data-based [28], are now tending to replace filtered-back projection algorithms. Other promising reconstruction algorithms, deep-learning-based Convolution Neural Network [29,30], enable the denoising of low-dose CT images while achieving a similar level of diagnostic quality. Numerous algorithms are being currently developed but still require clinical validation. Special attention should be paid to observer lesion detection performance at moderate and strong radiation dose reductions [31], enhancing the need to constrain the process on both radiation dosimetry and image quality aspects. Implementation of this process will facilitate the optimization steps by setting the next goal until reaching an ultra-low dose [32].

Finally, a number of limitations to this work must be mentioned. First, this process is based on the involvement of the teams. A multi-professional working group should periodically review the performance of each protocol to assess whether dose and image quality goals are being met and to consider next steps in the process. This means that time must be allocated in the team’s timetable to review the protocols. Secondly, the process was evaluated for chest CT only. Other anatomical locations should also be tested. Third, only a single radiologist was involved in image scoring, but this study was intended to be a feasibility study to evaluate the process. More than one radiologist should be involved in assessing image quality. Lastly, as we have seen with chest CT in obese patients, the CT process should be adapted to this patient profile.

## 5. Conclusions

In this study, we proposed a radiation dose optimization process for chest CT that could significantly reduce dose without compromising diagnosis.

## Figures and Tables

**Table 1:** Unenhanced chest CT acquisition and reconstruction parameters for the three modified acquisitions at high dose (HD), optimized dose (OD) and ultra-low dose (ULD) relative to the previous reference.

	Reference Protocol	HD Protocol		OD Protocol	ULD Protocol
Acquisition parameters					
Tube potential: Ref. kVp/Max. kVp/tissue cursor position	120/120/3	120/140/3		100/120/3	No kV_p_ modulation kV_p_ value = 100
Tube current: Quality ref. mAs/effective mAs	100/66	100/66		80/66	No mAs modulation mAs value = 10
Pitch/rotation time (s/rot)/CTDI_vol_ (mGy)	1.2/0.5/6.78	1.5/0.33/6.74		1.5/0.33/3.30	1.5/0.28/0.40
Slice thickness (mm)	1.5				
Reconstruction parameters					
Reconstruction 1: Mediastinum window					
Slice (mm)/increment (mm)/kernel			1.5/1.3/I31f		
Reconstruction 2: Lung window					
Slice (mm)/increment (mm)/kernel			1/0.8/I50f		

**Table 2:** Enhanced chest CT acquisition and reconstruction parameters according to two loops of the process.

	N_0_	N_1_	N_2_
Acquisition parameters			
Tube potential: Ref. kVp/kVp/tissue cursor position	100/100/9	100/100/11	80/100/11
Tube current: Quality ref. mAs/effective mAs	200/157		
Pitch/rotation time (s/rot)/CTDIvol (mGy)	1.5/0.33/5.63	1.3/0.33/6.50	1.4/0.285/4.65
Slice thickness (mm)	0.6	1	1
Reconstruction parameters			
Reconstruction 1: Mediastinum window			
Slice (mm)/increment (mm)/kernel	1/0.9/I26f		
Reconstruction 2: Lung window			
Slice (mm)/increment (mm)/kernel	1/0.9/I50f		

**Table 3:** Unenhanced chest CT dose metrics for the three protocols at high, optimized, and ultra-low doses compared to the reference protocol before optimization.

		DLP (mGy.cm)			CTDI_vol_ (mGy)			AD Variation Compared to Reference (in %)
Protocol	No. of Patients	LD	AD	DRL	LD	AD	DRL	
Reference	417	178.2	222.4	285.3	5.0	6.3	8.0	-
HD	49	214.1	298.6	361.3	5.7	8.3	10.6	+31.7
OD	367	99.0	130.7	188.7	2.7	3.7	5.3	−41.3
ULD	32	14.4	15.1	16.0	0.4	0.4	0.4	−93.6

Note: the LD, the AD, and the DRL are the 25th, 50th, and 75th percentiles of the distribution of median values of all dose data, respectively. Abbreviations: ULD: ultra-low dose; HD: high dose; OD: optimized dose; LD: low dose; AD: achievable dose; DRL: diagnostic reference level; DLP: Dose.Length Product; CTDI_vol_: Volume Computed Tomography Dose Index.

**Table 4:** Enhanced chest CT dose metrics according to two loops of the ALARA process, noted as N_1_ and N_2_. N_0_ represents the initial step of the process.

		DLP (mGy.cm)			CTDI_vol_ (mGy)			AD Variation Compared to N_0_ (in %)
	No. of Patients	LD	AD	DRL	LD	AD	DRL	
N_0_	564	174.1	281.1	336.9	5.0	8.1	9.6	-
N_1_	56	176.1	217.1	240.2	4.5	5.7	6.4	−29.6
N_2_	444	111.4	138.5	162.8	3.1	3.8	4.6	−53.1

Note: the LD, the AD, and the DRL are the 25th, 50th, and 75th percentiles of the distribution of median values of all dose data, respectively. Abbreviations: ULD: ultra-low dose; HD: high dose; LD: low dose; AD: achievable dose; DRL: diagnostic reference level; DLP: Dose.Length Product; CTDI_vol_: Volume Computed Tomography Dose Index.

## Data Availability

Data are available from the authors upon request.

## Supplementary Materials

The following supporting information can be downloaded at: https://www.mdpi.com/article/10.3390/jcm13164597/s1. Figure S1: Phantom study on the influence of Quality Reference mAs values on the CTDI_vol_ values according to three kV values in (a) and on the noise represented by the standard deviation of the Hounsfield Units measured in the lung in (b). Images were reconstructed with level 3 of the advanced modeled iterative reconstruction algorithm, and the medium-smooth mediastinum reconstruction kernel I31f was used. Table S1: Diagnostic reference levels in chest CT.
