# Supplementary material for: A Proposal for a Process from as Low as Reasonably Achievable to an Ultra-Low-Level Goal in Chest Computed Tomography
Source: J Clin Med. 2024 Aug 6;13(16):4597. doi: 10.3390/jcm13164597 (PMC11354269; doi:10.3390/jcm13164597)
Supplement: Supplementary file 1 [file jcm-13-04597-s001.zip › jcm-3099691-supplementary.pdf]

SUPPLEMENTAL MATERIALS

Figure S1. Phantom study on the influence of Quality Reference mAs values on the CTDIvol values according to three kV values in (**a**), and on the noise represented by the standard deviation of the Hounsfield Units measured in the lung in (**b**). Images were reconstructed with the level 3 of the advanced modeled iterative reconstruction algorithm with the medium smooth mediastinum reconstruction kernel I31f was used.

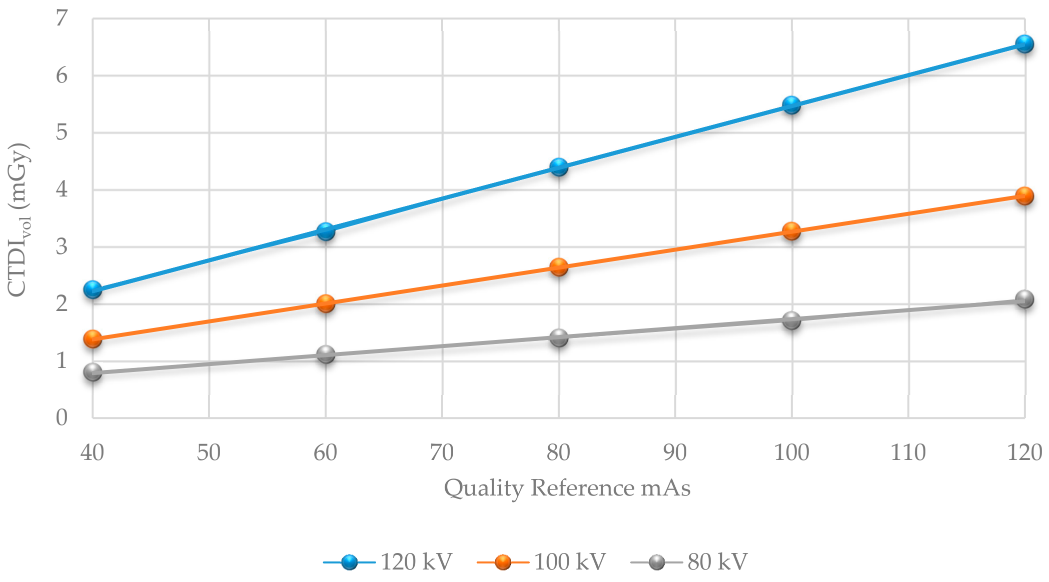

(**a**)

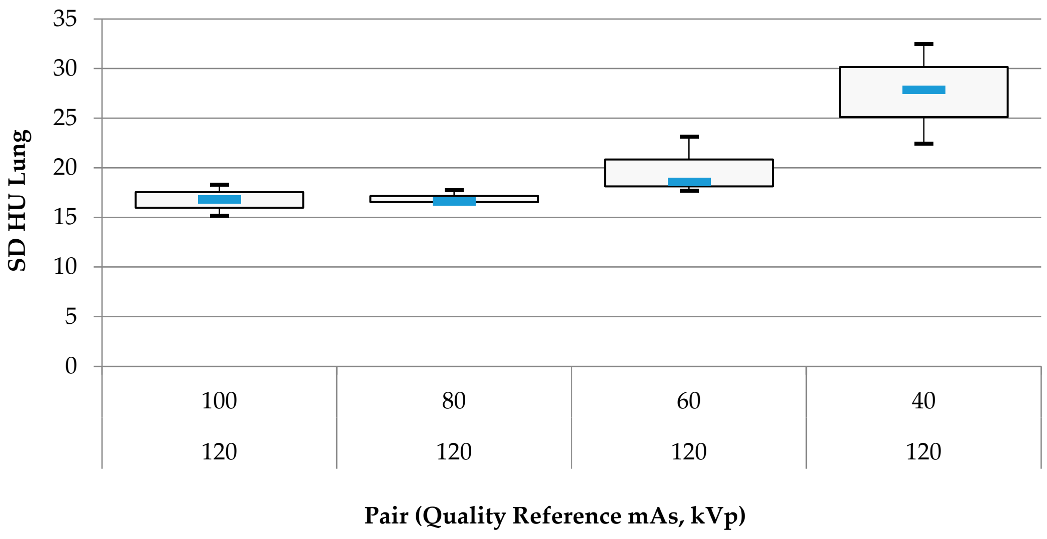

(**b**)

Abbreviations: SD: Standard Deviation; HU Lung: Hounsfield Units of lung insert; CTDIvol: Volume Computed Tomography Dose Index.

Table S1. Diagnostic reference levels in chest CT.

Clinical task	DLP (mGy.cm)	$CTDI_{vol}$ (mGy)
Unenhanced chest and Enhanced chest [10]	350	9,5
Lung cancer first and follow-up [4]	348	8
Detection of pulmonary embolism [4]	307	9
Unenhanced chest [5]	545	15
Enhanced chest [5]	596	16
Chest pulmonary arteries with contrast material [5]	557	18

Abbreviations: DRL: Diagnostic Reference level; DLP: Dose. Length Product; $CTDI_{vol}$: Volume Computed Tomography Dose Index.
